# Association of Kawasaki disease with intellectual disability, attention deficit hyperactivity disorder, and autism spectrum disorder: a systematic review and meta-analysis

**DOI:** 10.1186/s13052-025-01897-w

**Published:** 2025-02-21

**Authors:** Chih-Wei Hsu, Yu-Wei Lin, Yang-Chieh Brian Chen, Liang-Jen Wang, Ho-Chang Kuo

**Affiliations:** 1 Department of Psychiatry, Kaohsiung Chang Gung Memorial Hospital, Chang Gung University College of Medicine, Kaohsiung, Taiwan; 2 Department of Child and Adolescent Psychiatry, Kaohsiung Chang Gung Memorial Hospital, Chang Gung University College of Medicine, Kaohsiung, Taiwan; 3 Department of Pediatrics, Kaohsiung Chang Gung Memorial Hospital, Chang Gung University College of Medicine, Kaohsiung, Taiwan; 4 Kawasaki Disease Center, Kaohsiung Chang Gung Memorial Hospital, Kaohsiung, Taiwan

**Keywords:** ADHD, ASD, IQ, KD, Mental retardation

## Abstract

**Background:**

The relationship between Kawasaki disease (KD) and neurodevelopmental disorders (NDDs) remains unclear. This study aims to explore the association between them.

**Method:**

A systematic review was conducted using PubMed and Embase databases from inception to May 1, 2024 (INPLASY202450017). We included case-control or cohort studies comparing KD patients to healthy controls in assessing attention-deficit/hyperactivity disorder (ADHD), autism spectrum disorder (ASD), and intellectual disability (ID). The meta-analysis employed a random-effects model to calculate effect sizes using hazard ratios (HRs) with 95% confidence intervals (CIs) for the disease occurrence. Moreover, mean differences (MDs) were used to calculate intelligence quotient (IQ).

**Results:**

Four eligible studies, including 1,454,499 participants, were analyzed for ADHD, ASD, and ID. The risk of ADHD in KD patients was higher than in healthy controls (HR[95%CI] = 1.76[1.21–2.57]). However, the risks of ASD (HR[95%CI] = 1.68[0.47–5.94]) and ID (HR[95%CI] = 1.39[0.52–2.63]) were not significantly different between KD and controls. Additionally, three studies with 365 participants were analyzed for IQ. IQ comparisons showed no significant differences in full IQ (MD[95%CI]=-0.01[-2.44–2.42]), verbal IQ (MD[95%CI]=-1.05[-4.42–2.33]), and performance IQ (MD[95%CI]=-0.08[-2.75–2.59]).

**Conclusion:**

This study indicates that individuals with KD have a higher risk for ADHD but not for ASD or ID.

**Trial registration:**

INPLASY, INPLASY202450017. Registered 05 May 2024, https://inplasy.com/inplasy-2024-5-0017/.

**Supplementary Information:**

The online version contains supplementary material available at 10.1186/s13052-025-01897-w.

## Introduction

Kawasaki disease is the most prevalent vasculitis in children, predominantly affecting those under five years old, and it has an annual incidence of approximately 67 per 100,000 in this age group [1–3]. Clinical manifestations include conjunctivitis, mucosal changes (such as strawberry tongue), dermal changes, and cervical lymphadenopathy [3]. The most severe complications are cardiovascular, particularly coronary artery aneurysms. Non-cardiovascular complications include urinary and renal issues such as interstitial nephritis [4], intestinal obstruction [5], sensorineural hearing loss [6], and central nervous system sequelae [7].

The pathophysiology of central nervous system complications in Kawasaki disease is attributed to vasculitis and the deposition of cytokines and immune complexes [8, 9], likely due to a chronic inflammatory response. Immune and inflammatory responses also play significant roles in psychiatric disorders, with potential impact pathways including the complement system [10], infiltration of peripheral immune cells into the central nervous system [11], the gut-brain axis [12], and the Kynurenine Pathway [13]. Neuroimaging studies, such as single photon emission computed tomography (SPECT), have shown hypoperfusion in the bilateral cingulate gyri, thalamus, basal ganglia, brainstem, and cortex of the frontal lobes in Kawasaki disease patients with encephalitis [14]. In autism spectrum disorder (ASD), SPECT reveals hypoperfusion in the temporal regions involved in language processing and auditory perception, as well as the frontal regions related to executive function [15]. Additionally, Kawasaki disease [16] and attention-deficit/hyperactivity disorder (ADHD) [17, 18] are positively correlated with the occurrence of allergic diseases. These findings suggest potential associations between Kawasaki disease and neurodevelopmental disorders through immune and inflammatory responses. However, the relationship between Kawasaki disease and neurodevelopmental disorders, namely intellectual disability, ADHD, and ASD, remains unclear at the clinical level. Some studies suggest that Kawasaki disease increases the risk of these conditions [19–21], while others report no such association [22–25]. The current evidence is inconsistent. Given that these neurodevelopmental disorders can significantly impact individuals’ quality of life during adolescence and beyond, it is crucial to thoroughly investigate this relationship.

To address this knowledge gap, the present study aimed to conduct a meta-analysis to examine the association between Kawasaki disease and the risk of subsequent neurodevelopmental disorders, with a specific focus on common disorders such as intellectual disability, ADHD, and ASD. Moreover, this study also investigated differences in intelligence test scores between Kawasaki disease and healthy controls.

## Method

### Search strategy and selection criteria

This meta-analysis followed the guidelines outlined in the Meta-Analysis of Observational Studies in Epidemiology (MOOSE) statement (eTable 1) [26] and followed a predefined protocol registered on the International Platform for Registered Systematic Reviews and Meta-Analysis Protocols (INPLASY202450017). The Institutional Review Board of Chang Gung Memorial Hospital reviewed the study protocol and waived the need for ethics approval (Approval Number: 202200827B1). A systematic literature search was conducted from inception to May 1, 2024, using keywords (Kawasaki disease) AND (intellectual disability OR mental retardation OR cognition OR developmental delay OR ADHD OR autism OR ASD OR autistic disorder) in PubMed and Embase databases. Studies in all languages and regions were included. Detailed search strings are provided in eTable 2.

The inclusion criteria were as follows: (1) Cohort or case-control studies; (2) Studies including both participants diagnosed with Kawasaki disease and healthy controls; (3) Studies reporting the occurrence of intellectual disability, ADHD, and ASD among participants following their diagnosis of Kawasaki disease. Moreover, intellectual assessment measures such as intelligence tests were considered as additional indicators for cognitive outcomes. The exclusion criteria included: (1) Letters to the editor or editorial commentary; (2) Studies exclusively involving participants diagnosed with Kawasaki disease without healthy controls; (3) Studies reporting only symptom scale scores rather than diagnoses; (4) Studies potentially utilizing duplicate data, such as those from single-center, multicenter, or national registry studies conducted within the same country. Studies with smaller sample sizes were excluded as well. After screening titles and abstracts, eligible studies were subjected to full-text review to assess their suitability for inclusion. Two authors (LJW and YWL) independently conducted the review and study selection processes, with any discrepancies resolved through consultation with a third author (CWH).

### Data extraction and quality assessment

Data extraction was performed by two authors (CWH and LJW), including author names, publication year, study design, case numbers, and country. Primary outcomes were incidence of intellectual disability, ADHD, and ASD. Many studies utilized hazard ratios (HRs) as outcome measures for the risk of neurodevelopmental disorders [19, 23, 24], hence, HR data were extracted where available. For studies without relevant data, HR values were derived, for instance, by converting Kaplan-Meier survival curves. If cohort studies reported HR for different follow-up periods, the longest follow-up duration was selected. If studies adjusted HR for confounding covariates, the adjusted values ​​were preferred. Besides, intelligence assessment scales, as indicators of cognitive function, were also considered as alternative outcomes for intellectual disability in this study.

The risk of bias among included studies was independently assessed by two authors (YWL and CWH) using the Newcastle-Ottawa Scale’s risk of bias tool [27]. The Newcastle-Ottawa scale evaluates each study based on eight items across three domains, with a maximum score of 9 points. Studies scoring 7 to 9 points are considered good quality, those scoring 4 to 6 points are deemed fair quality, and those scoring 3 or lower are regarded as low quality. Discrepancies were resolved through consensus discussions involving a third author (LJW).

### Data synthesis and statistical analysis

Effect sizes were calculated using HRs with 95% confidence intervals (CIs) for the risk of intellectual disability, ADHD, and ASD. Mean differences (MDs) were employed for dichotomous outcomes such as intelligence test scores comparisons. Given potential heterogeneity among studies, a random-effects model was utilized for meta-analysis. Heterogeneity was assessed using the I-square statistic, categorized as low (≤ 25%), moderate (26–50%), or high (≥ 51%). If more than three studies were included, funnel plots and Egger’s tests were used to assess potential small study bias. Comprehensive Meta-Analysis software (version 4) was utilized for all analyses, with statistical significance set at a two-tailed *p*-value less than 0.05.

## Results

**Fig. 1:**
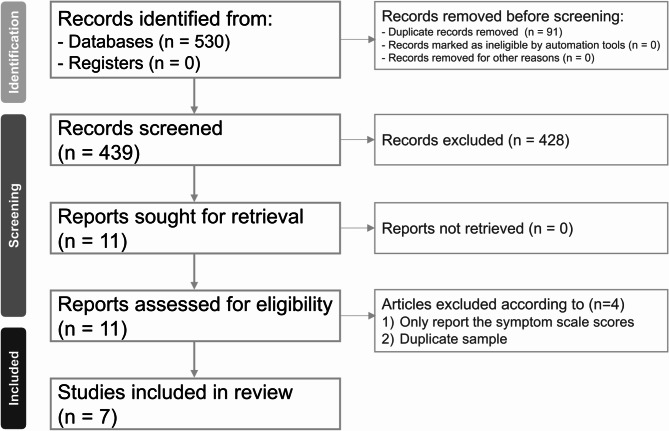
Flowchart of study selection

The study selection flowchart, adhering to the Preferred Reporting Items for Systematic Reviews and Meta-Analyses (PRISMA) guidelines, is presented in Fig. 1. A total of 530 studies were identified from databases. After removing duplicates and screening titles and abstracts, 11 studies remained. Subsequently, following full-text assessment, 4 studies were excluded for various reasons (eTable 3). The characteristics of the included studies are outlined in Table 1 [19, 22–25, 28, 29]. Four eligible studies comprised 518,621, 468,203, and 467,675 participants for aspects related to intellectual disability, ADHD, and ASD, respectively [19, 22–24]. Additionally, three eligible studies encompassed 365 participants for intelligence quotient assessment using the Wechsler Intelligence Scale for Children (WISC) [25, 28, 29]. Detailed quality assessments utilizing the Newcastle-Ottawa scale are provided in eTable 4. All included trials were adjudged to have fair or good quality.

**Table 1:** Characteristics of included studies

Topic	Study	Design	Case numbers	Female (%)	Disease onset age, year (range or mean)	Country	Newcastle-Ottawa scale
Intellectual disability							
	Wang (2018)	Cohort	Kawasaki disease: 4286 Healthy control: 50,038	1624 (38) 23,976 (48)	NA	Taiwan	9
	Robinson (2021)	Cohort	Kawasaki disease: 4597 Healthy control: 459,700	1834 (40) 183,400 (40)	0–18	Canada	8
Intelligence test							
	King (2000)	Cohort	Kawasaki disease: 32 Siblings control: 32	10 (31) 14 (44)	4–18	Canada	6
	Nishad (2010)	Cohort	Kawasaki disease: 20 Siblings control: 20	4 (20) 4 (20)	5–10	India	7
	Wang (2021)	Cross-section	Kawasaki disease: 176 Healthy control: 85	69 (39) 39 (46)	1.8	Taiwan	6
Attention deficit hyperactivity disorder							
	Kuo (2016)	Cohort	Kawasaki disease: 651 Healthy control: 3255	260 (40) 1300 (40)	0–10	Taiwan	9
	Robinson (2021)	Cohort	Kawasaki disease: 4597 Healthy control: 459,700	1834 (40) 183,400 (40)	0–18	Canada	8
Autism spectrum disorder							
	Kuo (2014)	Cohort	Kawasaki disease: 563 Healthy controls: 2815	222 (39) 1110 (39)	0–5	Taiwan	9
	Robinson (2021)^a^	Cohort	Kawasaki disease: 4597 Healthy control: 459,700	1834 (40) 183,400 (40)	0–18	Canada	8

^a^ Autism spectrum disorder was defined as a developmental disorder (no distinction) in the study by Robinson et al. Abbreviation: NA, not available

**Fig. 2:**
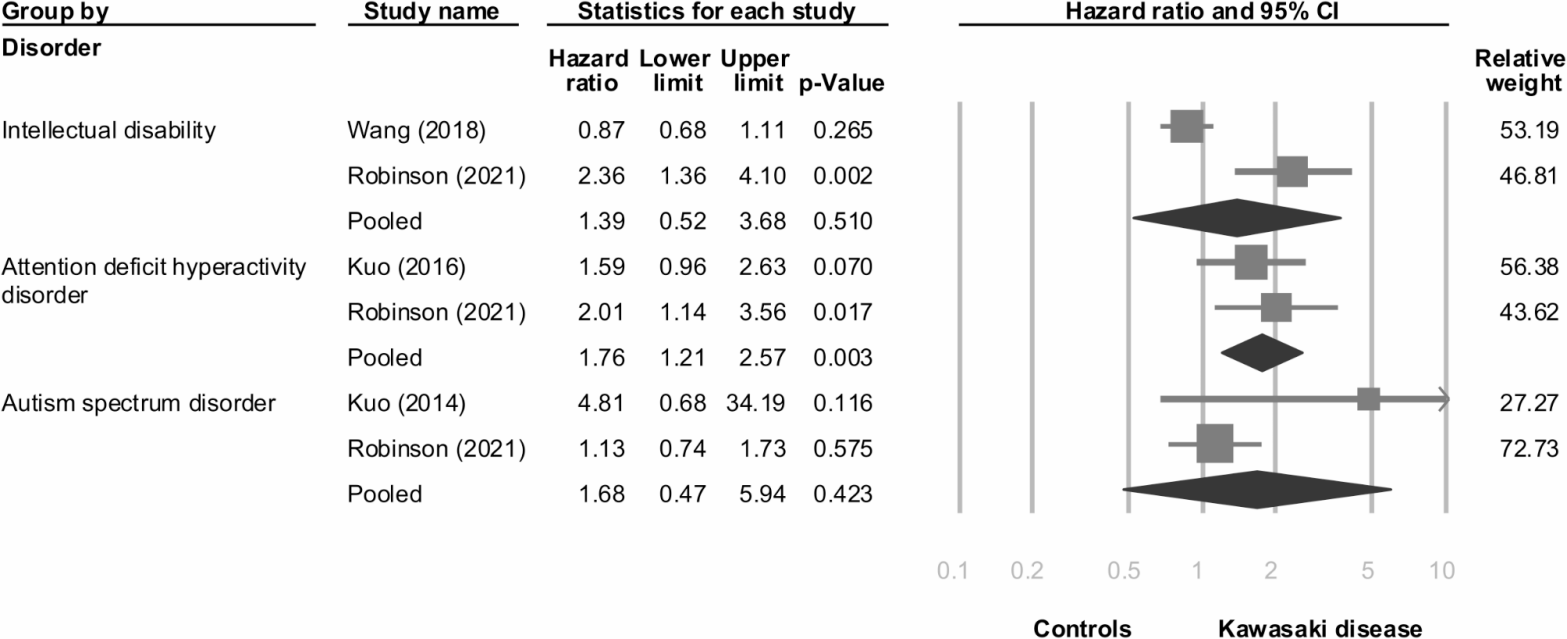
Forest plot of risk for intellectual disability, attention deficit hyperactivity disorder, and autistic spectrum disorder between Kawasaki disease and controls

Figure 2 illustrates the risks of intellectual disability, ADHD, and ASD in patients with Kawasaki disease compared to healthy controls. The risks of intellectual disability (HR [95%CI] = 1.39 [0.52 to 2.63]) and ASD (HR [95%CI] = 1.68 [0.47 to 5.94]) did not show statistically significant differences between Kawasaki disease and controls, with high heterogeneity for intellectual disability (I-square: 90%) and moderate heterogeneity for ASD (I-square: 50%). Furthermore, the risk of ADHD in Kawasaki disease (HR [95%CI] = 1.76 [1.21 to 2.57]) was higher than that of in controls, with low heterogeneity (I-square: 0%). However, due to the inclusion of only two studies, further examination through funnel plots and Egger’s tests was not feasible.

**Fig. 3:**
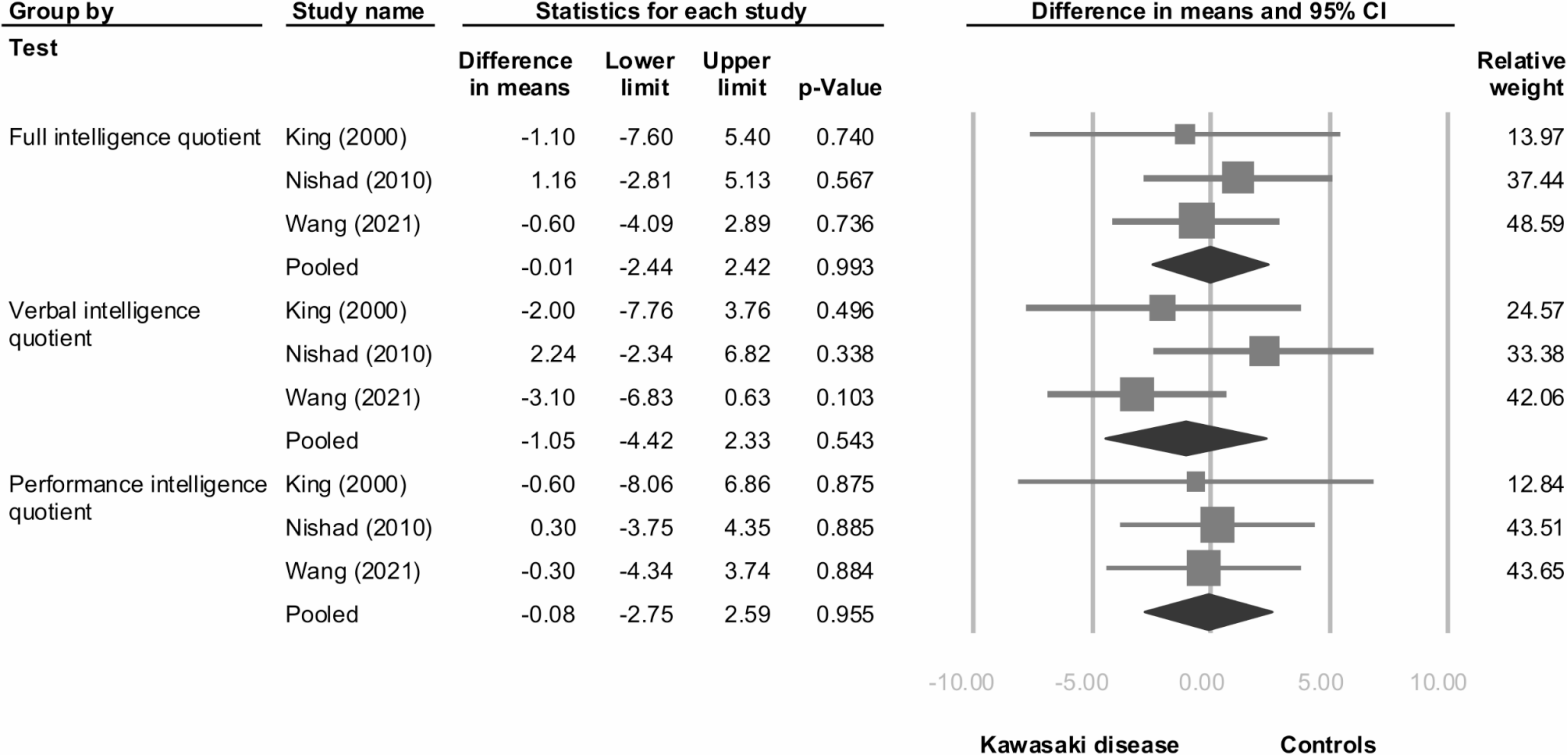
Forest plot of scale differences in full, verbal, and performance intelligence quotient between Kawasaki disease and controls

Figure 3 presents the differences in intelligence quotient scores measured by the WISC between Kawasaki disease and controls. No significant differences were observed in full intelligence quotient (MD [95%CI] = -0.01 [-2.44 to 2.42]), verbal intelligence quotient (MD [95%CI] = -1.05 [-4.42 to 2.33]), and performance intelligence quotient (MD [95%CI] = -0.08 [-2.75 to 2.59]). The heterogeneity among these outcomes ranged from low to moderate (I-square: full intelligence quotient = 0%; verbal intelligence quotient = 38%; performance intelligence quotient = 0%). The funnel plots for these outcomes are depicted in **eFigure 1**, revealing no significant bias upon examination.

## Discussion

To the best of our knowledge, this is the first meta-analysis to investigate whether Kawasaki disease elevates the risk of neurodevelopmental disorders. The study suggests that there is an increased risk of ADHD in individuals who had been diagnosed with Kawasaki disease compared to the general healthy population. However, Kawasaki disease does not contribute significantly to the risk of future intellectual disability or ASD. Furthermore, upon closer examination of cognitive function, Kawasaki disease seemed to have no significant effect on the differences in intelligence quotient test scores.

This study indicates that survivors of Kawasaki disease have a higher likelihood of being diagnosed with ADHD later on. ADHD has been consistently linked to neuroinflammatory states in many previous studies [30, 31], which aligns with relevant clinical research indicating that Kawasaki disease patients exhibit a decline in internalizing and attentional behavior during subsequent follow-ups [28]. Kawasaki disease has been found in past research to be associated with various immune disorders including atopic dermatitis, asthma, and allergic rhinitis [32], which stems from allergic reactions dominated by T Helper 2 cells and eosinophils [1, 33, 34]. ADHD has also been found to be associated with allergic diseases [35, 36]. In addition, ADHD has been linked to cellular and humoral immune responses, with higher detectable levels of IL-2, IL-5, IL-10, and TGF-beta in the cerebrospinal fluid [37] and higher serum concentrations of IL-6 and IL-10 in children with ADHD [38]. Furthermore, elevated levels of pro-inflammatory markers have been found to be positively correlated with the severity of ADHD symptoms [39]. These immune mechanisms may interact further with the dopamine system in the basal ganglia, potentially generating ADHD-related symptoms [40, 41]. Similar to ADHD, there is also ample evidence to suggest that the pathogenesis of Kawasaki disease is closely related to the immune system. The association of Kawasaki disease with cytokines including IL-6, IL-8, and IL-17, has been mentioned in several studies [1, 42, 43]. The development of coronary artery aneurysms in Kawasaki disease is also associated with the infiltration of neutrophils, macrophages, lymphocytes, and dendritic cells into the endothelial cells of blood vessels [44, 45]. Based on the aforementioned evidence, it can be understood that immune mechanisms are involved in the development of ADHD, as well as subsequent neurological and cognitive symptoms.

Our meta-analysis showed that Kawasaki disease does not significantly increase the risk of intellectual disability. This is contrary to previous studies which indicated that, in the Kawasaki disease population, reaching a level of systemic vasculitis may lead to reduced cerebral perfusion [46], potentially resulting in deficits in intelligence and cognitive function during development [47], and is also associated with a higher incidence of coronary artery lesions [46, 48]. However, previous cohorts have shown that Kawasaki disease patients who developed coronary artery lesions experienced no significant effects on their cognitive function [24]. In short, only a few cases of Kawasaki disease will develop severe central nervous system symptoms [7] or systemic vascular lesions [49], therefore not significantly increasing the risk of intellectual disability. Regarding the risk of ASD in the Kawasaki disease population, this study indicates no increased propensity. While past research suggested that ASD might be an immune disorder induced by allergies and associated with autoantibodies such as anti-myelin basic protein and anti-myelin associated glycoprotein [50], current meta-analysis evidence does not support an association between Kawasaki disease and an elevated risk of ASD. More evidence is needed to determine whether this immune response affects brain development and contributes to ASD.

Another important consideration is that the psychiatric sequelae observed in Kawasaki disease may involve mechanisms similar to severe acute respiratory syndrome coronavirus 2 (SARS-CoV-2)-induced post-infectious pediatric acute-onset neuropsychiatric syndrome (PANS) [51]. PANS is a condition affecting children and adolescents, characterized by an abrupt onset of neurological and psychiatric symptoms following infection [52]. A prior study indicated that PANS could be linked to ADHD and anxiety [53]. This is consistent with our findings, which suggest that the systemic inflammatory processes accompanying Kawasaki disease may create a neurobiological environment that fosters ADHD, but does not fully manifest as ASD or intellectual disability. However, this hypothesis requires further investigation to determine whether shared inflammatory and neuroimmune pathways underlie Kawasaki disease and conditions like SARS-CoV-2–related PANS.

This study has several limitations. First, research on the association between Kawasaki disease and neurodevelopmental disorders remains limited. The few studies that have been conducted have been conducted primarily in Taiwan and Canada, raising concerns about the generalizability of the findings across different racial and ethnic groups. However, it is worth noting that these studies utilized national-level databases and conducted long-term follow-up, providing valuable insights. Second, since the included studies relied on data from insurance databases, diagnoses of Kawasaki disease and neurodevelopmental disorders were based on diagnostic codes without clear documentation of the diagnostic instruments or tools used, potentially resulting in variations in the severity of these conditions that remain unknown. Third, the use of insurance databases also means that some potential confounders remain unaddressed. For example, there was insufficient information on family history of ADHD, ASD, intellectual disability, or other neurodevelopmental disorders. Although the included studies attempted to adjust for potential confounding factors by calculating adjusted hazard ratios (HRs), residual confounding cannot be ruled out. Finally, certain disease characteristics, such as gender, race, and age at the onset of Kawasaki disease, may influence the occurrence of subsequent outcomes. However, the current number of included studies is insufficient to conduct subgroup analyses.

## Conclusion

This study suggests that people with Kawasaki disease are at higher risk for ADHD, but the conclusions about risk of intellectual disability and ASD, or changes in intelligence quotient scores remain inconclusive. Moreover, insights from SARS-CoV-2-associated neuroinflammatory syndromes may help guide future research on the neurodevelopmental effects of Kawasaki disease.

## Electronic supplementary material

Below is the link to the electronic supplementary material.

Supplementary Material 1

## Data Availability

The data that support the findings of this study are available from the corresponding author, LJW and HCK, upon reasonable request.

## Abbreviations

ADHD: Attention-deficit/hyperactivity disorder
ASD: Autism spectrum disorder
CI: Confidence interval
HR: Hazard ratio
ID: Intellectual disability
IQ: Intelligence quotient
KD: Kawasaki disease
MD: Mean difference
MOOSE: Meta-analysis of observational studies in epidemiology statement
NDD: Neurodevelopmental disorder
PRISMA: Preferred reporting items for systematic reviews and meta-analyses guidelines
SPECT: Single photon emission computed tomography
WISC: Wechsler intelligence scale for children
