# Supplementary material for: Association of Kawasaki disease with intellectual disability, attention deficit hyperactivity disorder, and autism spectrum disorder: a systematic review and meta-analysis
Source: Ital J Pediatr. 2025 Feb 21;51:52. doi: 10.1186/s13052-025-01897-w (PMC11846269; doi:10.1186/s13052-025-01897-w)
Supplement: Supplementary file 1 — Supplementary Material 1 [file 13052_2025_1897_MOESM1_ESM.docx]

**Supplemental data**

**Association of Kawasaki disease with intellectual disability, attention deficit hyperactivity disorder, and autism spectrum disorder: a systematic review and meta-analysis**

Running Title: KD and neurodevelopmental disorders

Chih-Wei Hsu^a,1^, Yu-Wei Lin^a,1^, Yang-Chieh Brian Chen^a^, Liang-Jen Wang^b,#^, Ho-Chang Kuo^c,d,#^

^a^ Department of Psychiatry, Kaohsiung Chang Gung Memorial Hospital and Chang Gung University College of Medicine, Kaohsiung, Taiwan

^b^ Department of Child and Adolescent Psychiatry, Kaohsiung Chang Gung Memorial Hospital and Chang Gung University College of Medicine, Kaohsiung, Taiwan

^c^ Department of Pediatrics, Kaohsiung Chang Gung Memorial Hospital and Chang Gung University College of Medicine, Kaohsiung, Taiwan

^d^ Kawasaki Disease Center, Kaohsiung Chang Gung Memorial Hospital, Kaohsiung, Taiwan

^#^ Contributed equally as first author

^#^ Contributed equally as corresponding author and last author

^#^ Corresponding author:

Liang-Jen Wang, MD, PhD

Department of Child and Adolescent Psychiatry, Kaohsiung Chang Gung Memorial Hospital, Kaohsiung city, Taiwan

No. 123, Dapi Road, Kaohsiung, Taiwan

Tel.: 886-7-7317123 ext. 8753; Fax: 886-7-7326817

E-mail: wangliangjen@gmail.com

OR

Ho-Chang Kuo, MD, PhD

Department of Pediatrics, Kaohsiung Chang Gung Memorial Hospital and Chang Gung University College of Medicine, Kaohsiung, Taiwan

No. 123, Dapi Road, Kaohsiung, Taiwan

Tel.: 886-7-7317123 ext. 8320; Fax: +886-7-735-2225

E-mail: erickuo48@yahoo.com.tw or dr.hckuo@gmail.com

| **Content** | **Page** |
| --- | --- |
| eTable 1. MOOSE Checklist | 1 |
| eTable 2. Detailed search strategy | 2 |
| eTable 3. Excluded studies and reason | 3 |
| eTable 4. Detailed quality assessment of included studies using Newcastle-Ottawa scale | 4 |
| eFigure 1. Funnel plots in full, verbal, and performance intelligence quotient | 5-6 |
| References | 7 |

**eTable 1. MOOSE Checklist**

| **Item No** | **Recommendation** | **Reported on Page No** |
| --- | --- | --- |
| Reporting of background should include | | |
| 1 | Problem definition | 1-2 |
| 2 | Hypothesis statement | 1-2 |
| 3 | Description of study outcome(s) | 3-4 |
| 4 | Type of exposure or intervention used | 3 |
| 5 | Type of study designs used | 3 |
| 6 | Study population | 3 |
| Reporting of search strategy should include | | |
| 7 | Qualifications of searchers (eg, librarians and investigators) | 3 |
| 8 | Search strategy, including time period included in the synthesis and key words | 3, eTable 2 |
| 9 | Effort to include all available studies, including contact with authors | 3 |
| 10 | Databases and registries searched | 3 |
| 11 | Search software used, name and version, including special features used (eg, explosion) | N/A |
| 12 | Use of hand searching (eg, reference lists of obtained articles) | N/A |
| 13 | List of citations located and those excluded, including justification | 5, Table 1, eTable 3 |
| 14 | Method of addressing articles published in languages other than English | 3 |
| 15 | Method of handling abstracts and unpublished studies | 3 |
| 16 | Description of any contact with authors | N/A |
| Reporting of methods should include | | |
| 17 | Description of relevance or appropriateness of studies assembled for assessing the hypothesis to be tested | 3-4 |
| 18 | Rationale for the selection and coding of data (eg, sound clinical principles or convenience) | 3-4 |
| 19 | Documentation of how data were classified and coded (eg, multiple raters, blinding and interrater reliability) | 3 |
| 20 | Assessment of confounding (eg, comparability of cases and controls in studies where appropriate) | 4, eTable 4 |
| 21 | Assessment of study quality, including blinding of quality assessors, stratification or regression on possible predictors of study results | 4, eTable 4 |
| 22 | Assessment of heterogeneity | 4 |
| 23 | Description of statistical methods (eg, complete description of fixed or random effects models, justification of whether the chosen models account for predictors of study results, dose-response models, or cumulative meta-analysis) in sufficient detail to be replicated | 4 |
| 24 | Provision of appropriate tables and graphics | All tables, 15 |
| Reporting of results should include | | |
| 25 | Graphic summarizing individual study estimates and overall estimate | Figure 2-3 |
| 26 | Table giving descriptive information for each study included | Table 1 |
| 27 | Results of sensitivity testing (eg, subgroup analysis) | N/A |
| 28 | Indication of statistical uncertainty of findings | 5 |
| 29 | Quantitative assessment of bias (eg, publication bias) | eFigure 1 |
| 30 | Justification for exclusion (eg, exclusion of non-English language citations) | 5, eTable 3 |
| 31 | Assessment of quality of included studies | 4, eTable 4 |
| Reporting of conclusions should include | | |
| 32 | Consideration of alternative explanations for observed results | 7 |
| 33 | Generalization of the conclusions (ie, appropriate for the data presented and within the domain of the literature review) | 8 |
| 34 | Guidelines for future research | 7 |
| 35 | Disclosure of funding source | 9 |

*From*: Stroup DF, Berlin JA, Morton SC, et al, for the Meta-analysis Of Observational Studies in Epidemiology (MOOSE) Group. Meta-analysis of Observational Studies in Epidemiology. A Proposal for Reporting. *JAMA*. 2000;283(15):2008-2012. doi: 10.1001/jama.283.15.2008.

**eTable 2. Keywords and search results in different databases**

| **Database** | **Keyword** | **Filter** | **Date** | **Results** |
| --- | --- | --- | --- | --- |
| PubMed | (kawasaki disease) AND ((intellectual disability) OR (mental retardation) OR "cognition" OR (developmental delay) OR "ADHD" OR "autism" OR "ASD" OR (autistic disorder)) | Not applied | May 1, 2024 | 164 |
| Embase | kawasaki AND disease AND (intellectual AND disability OR (mental AND retardation) OR 'cognition' OR (developmental AND delay) OR 'adhd' OR 'autism' OR 'asd' OR (autistic AND disorder)) | All field | May 1, 2024 | 366 |

**eTable 3. Excluded studies and reasons**

| Reasons | Reference |
| --- | --- |
| Only report the symptom scale scores | 1. 55th Annual Meeting of the Association for European Paediatric and Congenital Cardiology (AEPC) Geneva, Switzerland \| 25–28 May 2022." Cardiology in the Young 32(S2): S1-S279. 2. Fauteux, A. A., R. Gutierrez Rojas, K. Agbogba, M. Benovoy, A. R. Charlebois-Poirier, E. Lalancette, S. Lippe and N. Dahdah (2023). "Electroencephalography signals and neurodevelopment after Kawasaki disease: A pilot study." Pediatr Int 65(1): e15482. |
| Duplicate sample | 1. Lin, C. H., W. D. Lin, I. C. Chou, I. C. Lee and S. Y. Hong (2019). "Heterogeneous neurodevelopmental disorders in children with Kawasaki disease: what is new today?" BMC Pediatr 19(1): 406. 2. Chen, D. T., J. P. Chang, S. W. Cheng, H. C. Chang, J. H. Hsu, H. H. Chang, W. C. Chiu and K. P. Su (2022). "Kawasaki disease in childhood and psychiatric disorders: A population-based case-control prospective study in Taiwan." Brain Behav Immun 100: 105-111. |

**eTable 4. Study quality assessed using the Newcastle-Ottawa scale**

|  | **Selection** | | | | **Comparability** | **Outcome** | | | **Total** |
| --- | --- | --- | --- | --- | --- | --- | --- | --- | --- |
|  | Representativeness of patients | Selection of control | Ascertainment of exposure | Interest outcome did not present |  | Assessment of outcome | Sufficient follow-up | Adequate follow-up |  |
| Wang (2018)^1^ | 1 | 1 | 1 | 1 | 2 | 1 | 1 | 1 | 9 |
| Robinson (2021)^2^ | 1 | 1 | 1 | 0 | 2 | 1 | 1 | 1 | 8 |
| King (2000)^3^ | 0 | 1 | 1 | 0 | 2 | 1 | 0 | 1 | 6 |
| Nishad (2010)^4^ | 1 | 1 | 1 | 1 | 1 | 1 | 0 | 1 | 7 |
| Wang (2021)^5^ | 1 | 1 | 1 | 0 | 1 | 1 | 0 | 1 | 6 |
| Kuo (2016)^6^ | 1 | 1 | 1 | 1 | 2 | 1 | 1 | 1 | 9 |
| Kuo (2014)^7^ | 1 | 1 | 1 | 1 | 2 | 1 | 1 | 1 | 9 |

**eFigure 1. Funnel plots in full, verbal, and performance intelligence quotient**

**(A) Full intelligence quotient**

**(B) Verbal intelligence quotient**

**(C) Performance intelligence quotient**

**References**

1. Wang LJ, Kuo HC. Cognitive Development After Kawasaki Disease　- Clinical Study and Validation Using a Nationwide Population-Based Cohort. *Circ J*. Jan 25 2018;82(2):517-523. doi:10.1253/circj.CJ-17-0557

2. Robinson C, Lao F, Chanchlani R, Gayowsky A, Darling E, Batthish M. Long-term hearing and neurodevelopmental outcomes following Kawasaki disease: A population-based cohort study. *Brain Dev*. Aug 2021;43(7):735-744. doi:10.1016/j.braindev.2021.03.001

3. King WJ, Schlieper A, Birdi N, Cappelli M, Korneluk Y, Rowe PC. The effect of Kawasaki disease on cognition and behavior. *Arch Pediatr Adolesc Med*. May 2000;154(5):463-8. doi:10.1001/archpedi.154.5.463

4. Nishad P, Singh S, Sidhu M, Malhi P. Cognitive and behaviour assessment following Kawasaki disease--a study from North India. *Rheumatol Int*. Apr 2010;30(6):851-4. doi:10.1007/s00296-009-1078-1

5. Wang LJ, Tsai ZY, Chang LS, Kuo HC. Cognitive development of children with Kawasaki disease and the parenting stress of their caregivers in Taiwan: a case-control study. *BMJ Open*. Jun 3 2021;11(6):e042996. doi:10.1136/bmjopen-2020-042996

6. Kuo HC, Chang WC, Wang LJ, Li SC, Chang WP. Association of Attention deficit hyperactivity disorder and Kawasaki disease: a nationwide population-based cohort study. *Epidemiol Psychiatr Sci*. Dec 2016;25(6):573-580. doi:10.1017/s2045796015000840

7. Kuo HC, Wu CM, Chang WP, et al. Association between Kawasaki disease and autism: a population-based study in Taiwan. *Int J Environ Res Public Health*. Apr 3 2014;11(4):3705-16. doi:10.3390/ijerph110403705
